# New Biomarkers in Cancers

**DOI:** 10.3390/cancers13040708

**Published:** 2021-02-09

**Authors:** Daniel Novak, Jochen Utikal

**Affiliations:** 1 Skin Cancer Unit, German Cancer Research Center (DKFZ), 69121 Heidelberg, Germany; d.novak@dkfz-heidelberg.de; 2 Department of Dermatology, Venereology and Allergology, University Medical Center Mannheim, Ruprecht-Karl University of Heidelberg, 68167 Mannheim, Germany

In this Special Issue of *Cancers,* the latest insights on biomarkers in cancers are presented in 33 up-to-the-minute research papers and reviews summing up the tremendous progress in this interesting and important field of research. The recent development of new therapeutic approaches has provided clinicians with more efficient tools than ever before for the treatment of cancerous diseases. However, choosing the right option requires to precisely determine the type and stage of disease. Moreover, routinely diagnosing cancer at the earliest stage possible would greatly enhance the beneficial effects of established cancer therapies and drastically increase the patients’ chances for complete recovery.

Biomarkers serve as valuable diagnostic indicators that can help clinicians to ascertain what type of cancer they are dealing with, how far a particular cancer has progressed, and what kind of molecular dysregulation might have caused the outbreak of the disease. Typical biomarkers are quantifiable parameters such as molecules, mutations or biological processes. This Special Issue of *Cancers* covers different topics related to biomarkers, demonstrating their diversity.

First, there are the more classical markers represented by larger biomolecules, such as proteins. Flieswasser and colleagues report on establishing a validated immunohistochemistry (IHC) staining for the detection of CD70, a marker commonly expressed in solid and hematological tumor entities [1]. Another IHC study investigated the expression of the melatonin receptors MT1 and MT2 in non-small cell lung cancer (NSCLC) showing lower expression in more advanced stages of the disease and concluding that MT2 especially could serve as a prognostic marker [2]. Banys-Paluchowski et al. quantified the levels of collagen degradation markers (CDMs) upon neoadjuvant chemotherapy of breast cancer patients, reasoning that CDMs could be of diagnostic importance [3]. A study on the applicability of serum cancer antigen 72-4 (CA72-4) as a predictive marker for gastric cancer points out that it could be useful in combination with esophagogastroduodenoscopy (EGD) despite having a low predictive value on its own [4]. In a similar fashion, Lee et al. demonstrated that measuring washout cytokeratin fragment 21-1 (CYFRA 21-1) together with washout thyroglobulin (Tg) and fine needle aspiration cytology (FNAC) increases the sensitivity of metastatic lymph node evaluation compared to FNAC and Tg washout alone, making these techniques more reliable [5]. Additionally, included in this Special Issue of *Cancers* are two informative reviews on CD147 and neuromedin U as tumor markers and potential therapeutic targets [6,7,8].

Another thematic focus in this Special Issue addresses cells as biomarkers discussing tumor-associated circulating endothelial cells (tCECs) as a new diagnostic marker for prostate cancer [9], the detailed quantification of leukocytes in peripheral blood to distinguish head and neck squamous cell carcinoma cancer patients from healthy individuals [10], the utility of peripheral blood mononuclear cells (PBMCs) as a marker to assess the response of NSCLC patients to nivolumab therapy [11] and the development of a highly efficient technique that allows the detection of circulating tumor cells in blood samples from NSCLC patients [12]. Moreover, Payne et al. review the status quo of diagnosing head and neck cancer with the help of circulating tumor cells and cast a glance at potential new applications of this method [13].

miRNAs are a class of molecules that have attracted a lot of attention in recent years. Their involvement in many pathophysiologic processes is reflected by numerous publications showing that they could serve as tumor markers. Here, we present a couple of new studies that report on miRNAs as biomarkers of different tumor entities [14,15,16,17]. Moreover, the usability of miRNAs as diagnostic and predictive markers is reviewed by Machakova et al. and Royam et al. [18,19].

In search of markers and methods that allow for highly accurate and sensitive diagnosis of cancer type and staging, the analysis of methylation patters might prove useful. While Leiro-Fernandez et al. report that methylation marker analysis significantly improves mediastinal staging of lung cancer patients [20], the work of Ibrahim and colleagues indicates that ascertaining the methylation marks of the Gasdermin E gene allows to detect a wide variety of cancer types and, at the same time, facilitates to distinguish between different cancer entities, thus making diagnosis much more precise [21].

Trying to identify biomarkers suitable for the diagnosis of cancerous diseases usually requires the processing of large quantities of samples and data. Computational and high-throughput methods have become more and more relevant and will enable scientists to find markers that are more useful in less time. Cesselli et al. describe the use of an artificial intelligence algorithm analyzing and combining diagnostic data obtained with established methods in order to assess the prognosis of grade II glioma patients [22]. Another study utilized network analyses of multiple myeloma (MM) datasets and demonstrated that this approach could prove advantageous in linking specific drug response patterns to different molecular characteristics of different MM phenotypes [23]. Menyhart and colleagues screened the transcriptomic profiles of colorectal cancer specimens with reference to specific driver mutations and thereby detected potential targets that could serve as attack points for anticancer drugs [24]. The benefits of omics-based approaches in managing the variability of breast cancers is reviewed by Saini et al., who pointed out that methods that interrelate large quantities of data from different sources will tremendously broaden our knowledge of tumor biomarkers [25].

Tumor biomarkers are not only helpful for diagnosing and staging patients but for follow-up monitoring and evaluating if a certain patient has a good or bad prognosis post treatment. In this Special Issue, a non-invasive method of examining the urinary metabolome of bladder cancer patients with the help of nuclear magnetic resonance (NMR) is described [26]. Furthermore, a histologic study on prostate cancer samples revealed that a low grade of vascularization is an indicator for a more aggressive tumor and a poorer prognosis [27].

It is quite evident that traditional and newly found biomarkers are not mutually exclusive tools but complement each other. Together with the employment of state-of-the-art technology, these tools will provide clinicians with reliable and efficient options for diagnosing cancer.

This Special Issue of *Cancers* also features additional informative reviews on a broad range of tumor-marker-related subjects. Established and promising potential biomarkers for colorectal cancer [28], as well as neuroendocrine gastroenteropancreatic tumors, are discussed here [29]. Shindo et al. and Otoshi et al. summarize the current knowledge about tumor markers and cancer immunotherapy and address the need for novel biomarkers that would help to predict if a certain patient would respond to a specific type of immunotherapy [30,31]. A view on two different technical aspects of tumor biomarker detection is given by Luchini et al. and Catino and colleagues who report on liquid biopsy and its benefits for diagnosing pancreatic cancer and the potential of breath analysis for early diagnosis of malignant pleural mesothelioma [32,33].

In conclusion, this Special Issue illustrates that research on tumor biomarkers is a very diverse and complex field of research. Despite the advances that have been made in recent years, there is still a lot to discover and to understand. However, the success of cancer therapies is strongly linked to finding diagnostically conclusive tumor biomarkers. For this reason, it is essential to push this field of research forward.
